# Balloon-Assisted Coronary Intravascular Lithotripsy for Large Severely Calcified Coronary Artery Stenosis

**DOI:** 10.1016/j.jscai.2024.101348

**Published:** 2024-03-06

**Authors:** Brett Yarusi, Mohammad Hashim Mustehsan, Sripal Bangalore

**Affiliations:** Department of Medicine, Leon H. Charney Division of Cardiology, NYU Grossman School of Medicine, New York, New York

**Keywords:** coronary artery disease, intravascular lithotripsy, intravascular ultrasound, myocardial infarction

## Abstract

We present a patient with in-stent restenosis due to severe coronary calcification with asymmetric stent expansion and resulting stent eccentricity in a very large (6.0 mm) caliber coronary artery. We demonstrate the feasibility of using the largest commercially available coronary intravascular lithotripsy balloon (4.0 mm) along with a “buddy” balloon inflated simultaneously to treat focal coronary artery calcification in a vessel with a diameter significantly larger than the largest commercially available coronary intravascular lithotripsy balloon. To our knowledge, this is the first demonstration of this technique in coronary artery intervention.

## Case presentation

A 59-year-old Jehovah’s Witness man presented to the emergency department with progressively worsening substernal chest discomfort, for 2 weeks prior to presentation. His medical history included hypertension, hyperlipidemia, end-stage renal disease (ESRD) on hemodialysis, hyperparathyroidism with surgery in 2001 with focal recurrent adenoma, and severe multivessel coronary artery disease first diagnosed in November 2021 during an admission for non–ST segment elevation myocardial infarction (NSTEMI). He was found to have very large caliber coronaries with severe calcification and chronic total occlusion (CTO) of the mid-left anterior descending (LAD) artery, mid-right coronary artery, and severe stenosis of the ramus. He was deemed not to be a surgical candidate and subsequently underwent intravascular ultrasound (IVUS)-guided CTO percutaneous coronary intervention (PCI) of LAD and ramus, both treated with rotational atherectomy and placement of drug-eluting stents (DES). Ten months later, he presented with recurrent NSTEMI and was found to have severe restenosis of the proximal edge of the mid-LAD stent. IVUS showed 360° calcification of the proximal and mid-LAD with areas of eruptive calcific nodule in the proximal LAD. He underwent PCI with cutting balloon atherectomy, followed by Shockwave intravascular lithotripsy (IVL), and a 5.0 mm drug-eluting stent, which was postdilated to 6.0 mm with a noncompliant (NC) balloon to high pressure, with excellent angiographic results. The final minimal stent area was >15 mm^2^. Cilostazol was added in addition to dual antiplatelet therapy with aspirin to reduce the risk of restenosis but was discontinued soon after owing to side effects.

**Figure 1:**
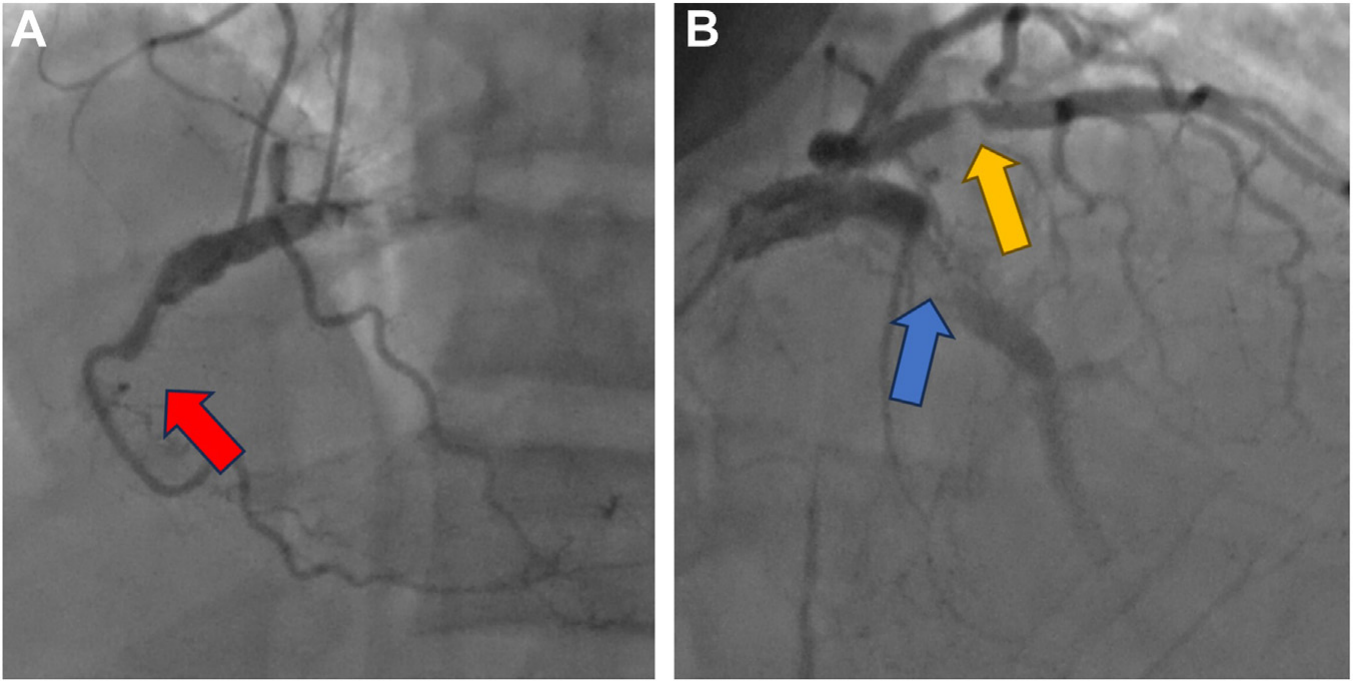
**Preinterventional angiogram.** (**A**) The right coronary artery chronic total occlusion (left, red arrow); (**B**) severe left anterior descending artery in-stent restenosis (right, blue arrow) and severe ramus in-stent restenosis (right, yellow arrow).

During his current presentation, initial laboratory studies were notable for an elevated high-sensitivity troponin-T to 938 ng/L consistent with NSTEMI. Echocardiogram showed an ejection fraction (EF) of 30%, which was newly reduced from 60%. Urgent coronary angiography demonstrated severe multivessel coronary artery disease with CTO of the mid-right coronary artery (unchanged), a severely calcified in-stent restenosis (ISR) CTO of a previously implanted mid-LAD DES (at a site proximal to the 2 layers of stent implanted previously), and severe ISR of the previously implanted mid-ramus intermedius DES (Figure 1). Given these findings, the patient was again referred for consideration of surgical revascularization. However, he was deemed to have very high surgical risk given calcified aorta, calcified coronaries (with concern of inability to find a noncalcific part to sew the graft), low EF, and ESRD and being a Jehovah’s Witness. The decision was therefore made to proceed with complex PCI to the mid-LAD ISR CTO and the severe mid-ramus ISR.

**Figure 2:**
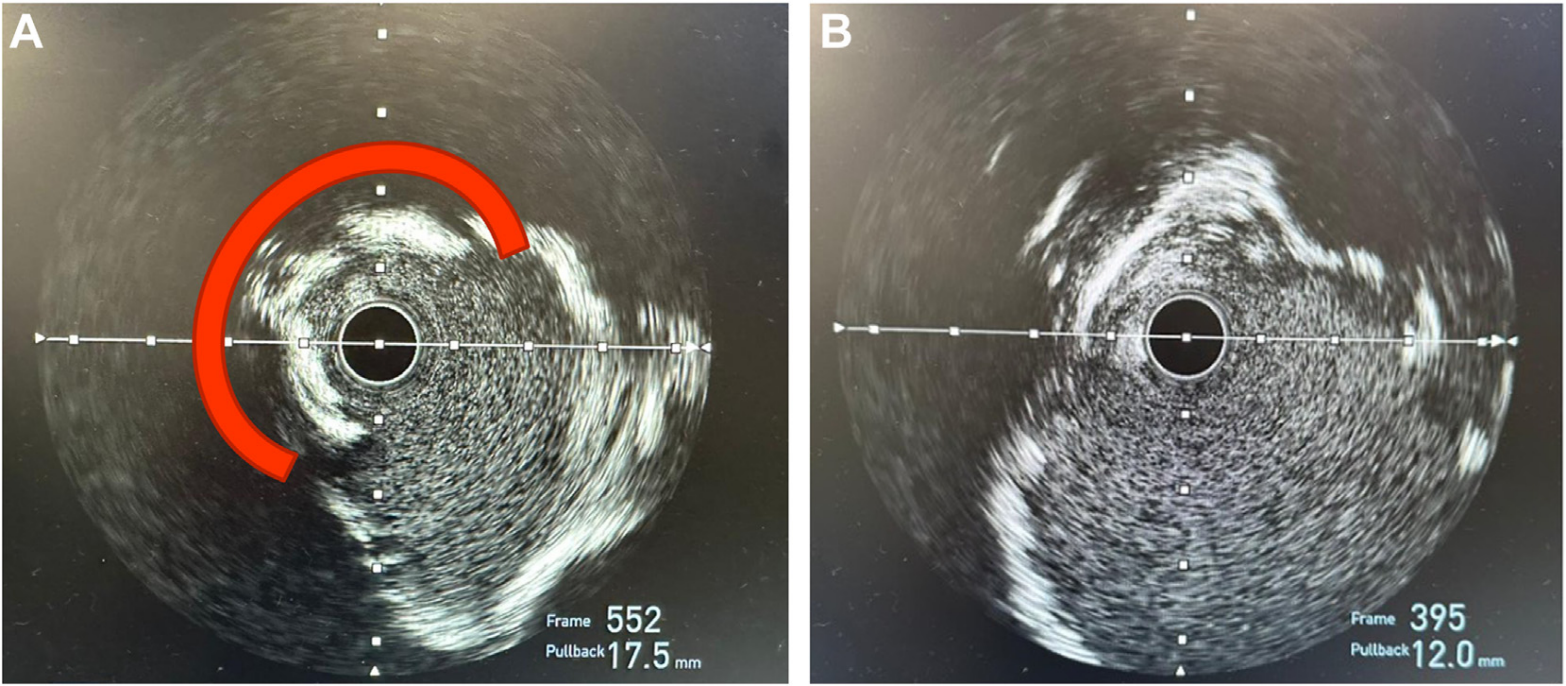
**Intravascular ultrasound images.** (**A**) Preinterventional intravascular ultrasound with a >180° arc of unfractured calcification (red). (**B**) Postinterventional intravascular ultrasound demonstrating significant improvement in-stent eccentricity.

**Figure 3:**
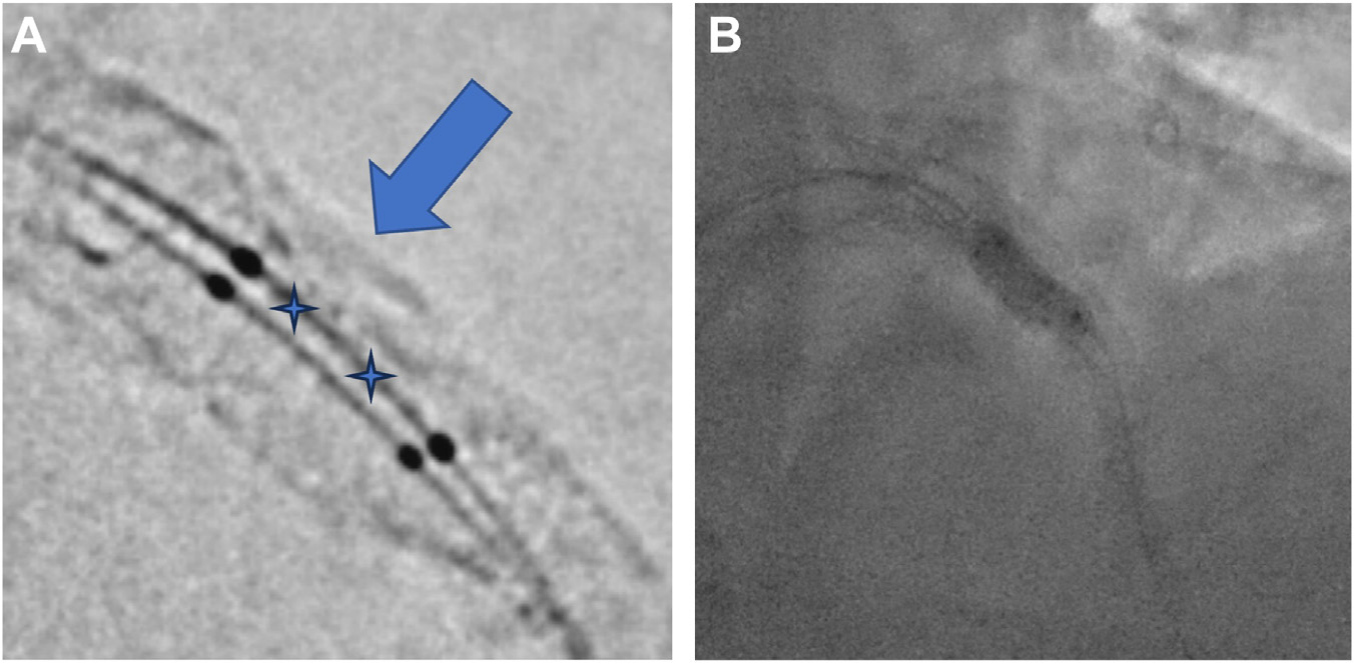
**Buddy shock technique.** (**A**) Positioning of the intravascular lithotripsy balloon (stars showing location of lithotripsy emitters) adjacent to the calcific nodule (arrow) with a buddy balloon. (**B**) Kissing inflation of the intravascular lithotripsy balloon with the noncompliant buddy balloon.

In the cardiac catheterization laboratory, arterial access was obtained in the left distal radial artery with a short 7F sheath. The left main coronary artery was engaged with a 7F EBU 4.5 Launcher Guide (Medtronic). Predilation of the mid-LAD ISR CTO was then performed with a 2.0-mm compliant balloon. Following predilation, IVUS of mid-LAD ISR demonstrated diffuse 360° calcification of the proximal and mid LAD. At the site of the ISR, although the 360° calcification was fractured in part (from the previous procedure IVL and stent implantation), there remained a focal region of stent underexpansion and eccentricity due to severe circumferential unfractured native calcification with an associated calcific nodule with a reference vessel diameter of 6.0 mm (Figure 2). Further predilation with high pressure 4.0 mm cutting balloon and serial NC balloons including a 5.0 Sapphire NC balloon (Cardiovascular Systems, Inc) inflated to a maximum pressure of 24 atm were performed. Repeat IVUS showed persistence of the unfractured arc of calcium and stent eccentricity (Figure 2). Given these IVUS findings despite upfront lesion modification with serial high-pressure NC balloons, the decision was made to proceed with IVL. A 4.0-mm × 12.0-mm Shockwave C2+ IVL Balloon Catheter (Shockwave Medical) was positioned directly adjacent to the region of stent underexpansion within the previously implanted mid-LAD DES. A 3.5-mm × 12.0-mm NC Euphora NC balloon (Medtronic) was subsequently positioned directly adjacent to the Shockwave C2+ IVL balloon (Figure 3). The 2 balloons were then simultaneously inflated to 4 atm, with excellent apposition of the IVL balloon to the focal region of stent underexpansion on fluoroscopy (verified on a stent boost mode). A total of 80 IVL pulses were subsequently delivered in 10 pulse series, with simultaneous deflation and reinflation of the 2 balloons performed between each series of therapy. The lesion was further predilated with a 6.0- × 8.0-mm NC EMERGE balloon (Boston Scientific). Treatment with a peripheral drug-coated balloon (off-label) using a 4.0-mm × 40-mm Ranger Drug-Coated Balloon (Boston Scientific) was attempted. However, despite the use of a buddy wire and guide extension, the balloon could not be delivered. A 5.0-mm × 16.0-mm DES was then successfully implanted with postdilation with the same 6.0 NC balloon to 24 atm. IVUS performed after stenting showed a well-apposed DES, with a minimal stent area of 24.1 mm^2^ and resolution of stent eccentricity (Figure 3).

## Discussion

**Figure 4:**
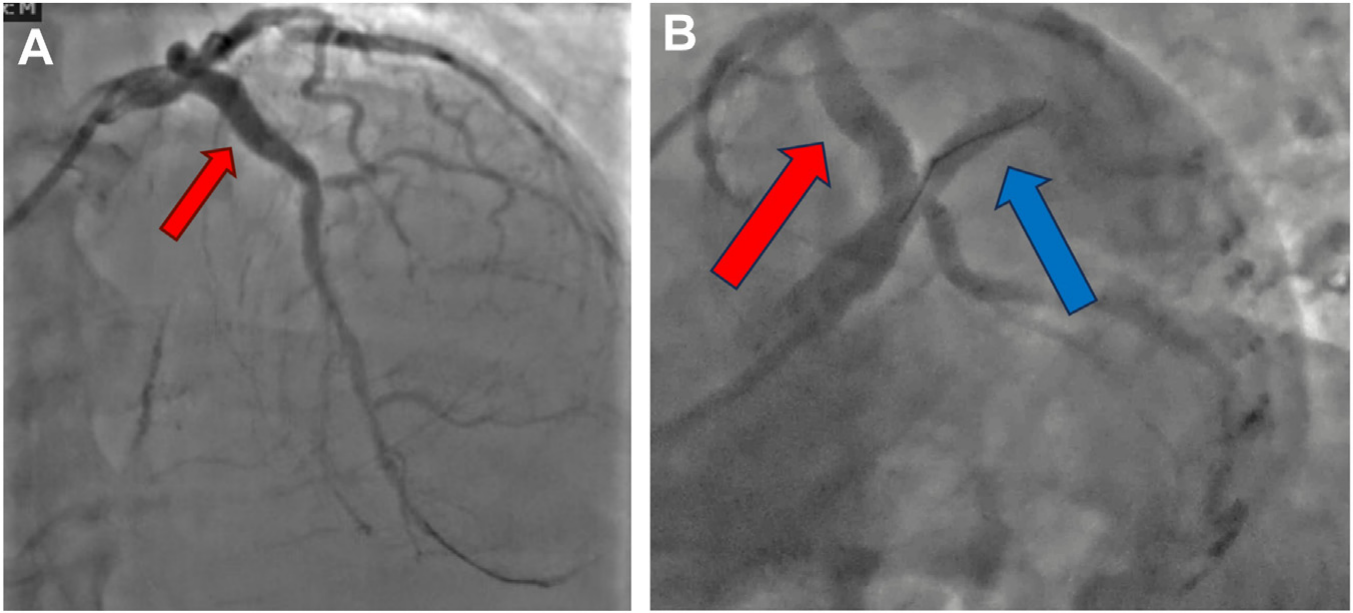
**Postinterventional angiogram.** (**A**) Well-expanded stents in the left anterior descending artery (red arrow). (**B**) Well-expanded stent in the ramus coronary (blue arrow) artery.

Suboptimal stent expansion and stent eccentricity have been shown to be strong predictors of stent failure.^1^ This is thought to be secondary to disruption of laminar flow and nonhomogenous drug distribution from eccentric stent struts.^1^ In this report, we present the case of a patient with severe restenosis in a large (6.0-mm diameter) caliber LAD, which was previously treated with rotational atherectomy and a 4.0-mm Shockwave IVL with the placement of a large DES postdilated with a 6.0-mm balloon to high pressure with a resulting large minimal stent area. IVUS performed to elucidate the etiology of restenosis showed stent eccentricity due to focal unfractured area of the 360° calcification and a calcific nodule. Although nonstent–related factors (ESRD and hyperparathyroidism) was also considered as part of the etiology for the ISR, the focal nature of ISR pointed to stent eccentricity as the most probable cause; however, it was likely multifactorial. Given the large caliber of the vessel, the largest commercially available coronary IVL balloon (4.0 mm) would have been ineffective in isolation due to the size of the vessel. Moreover, it was felt that even with the largest burr, rotational atherectomy will be biased toward the inner curvature of the artery (unfractured calcification and eccentricity was on the outer curvature) and therefore would not be effective. Peripheral IVL was deemed to be too long for the lesion to be treated. Furthermore, the same lesion had previously been treated with a 4.0 mm IVL, rotational atherectomy and high-pressure NC balloon. Recognizing this history, we decided to proceed with a novel approach of pairing the IVL balloon with a “buddy” balloon to ensure adequate contact with the calcification. We used a 3.5-mm NC balloon paired with a 4.0-mm IVL balloon to have a combined diameter of 5.0 mm, which was slightly larger than 1:1 for the luminal diameter (vessel diameter = [2/3 × balloon diameter 1] + [2/3 × balloon diameter 2]). Using StentViz, we were able to ensure adequate apposition of the IVL balloon to the calcific nodule. After delivering IVL, the calcification yielded, and we were able to achieve an excellent result, both angiographically (Figure 4) and by IVUS with restoration of centricity of the stent. To our knowledge, this is the first use of this technique in coronary interventions. At 3-month follow up, the patient’s EF improved to 60% and he remains angina free. He successfully underwent surgical resection of the hyperparathyroid adenoma. He is maintained on phosphate binders to keep phosphorus goals between 3.5 and 5.0 mg/dL, with strict monitoring of parathyroid hormone and is on sevelamer to maintain calcium levels within the normal range.

## Conclusion

In this case report, we demonstrate the feasibility of using IVL with a buddy balloon to correct DES eccentricity due to severe unfractured coronary artery calcification in a vessel with a larger diameter than commercially available coronary IVL balloon. To our knowledge, this is the first demonstration of this technique in coronary artery intervention.
